# Prediabetes is associated with loss of appendicular skeletal muscle mass and sarcopenia

**DOI:** 10.3389/fnut.2023.1109824

**Published:** 2023-03-01

**Authors:** Shuying Li, Jiangfeng Mao, Weihong Zhou

**Affiliations:** ^1^Nanjing Drum Tower Hospital, The Affiliated Hospital of Nanjing University Medical School, Nanjing, Jiangsu, China; ^2^Peking Union Medical College Hospital, Chinese Academy of Medical Sciences, Peking Union Medical College, Beijing, China

**Keywords:** sarcopenia, prediabetes, NHANES, appendicular skeletal muscle mass, dual-energy X-ray absorptiometry

## Abstract

**Background:**

Decreasing mass and metabolism in skeletal muscle are associated with increasing insulin resistance (IR) and type 2 diabetes mellitus (T2DM). The causal relation between sarcopenia and abnormal glucose metabolism may be bidirectional. This investigation is aimed to explore the detailed correlation between pre-diabetes and sarcopenia in United States (US) adults.

**Methods:**

A total of 22,482 adults aged ≥20 years in the National Health and Nutrition Examination Survey (NHANES) were included. Generalized linear models were conducted to examine associations between diabetes status, serum glucose, glycohemoglobin (HbA1c), and sarcopenia. Generalized additive models and smooth fitting curves were used to examine the non-linear relationship between HbA1c and ASM_BMI_. Sarcopenia was defined as ASM_BMI_ (appendicular skeletal muscle mass/body mass index) < 0.789 for males, and <0.512 for females based on the cut-off values of the Foundation for the National Institutes of Health (FNIH) Sarcopenia Project.

**Results:**

After fully adjusting for multiple covariates, sarcopenia was directly correlated with pre-diabetes [OR (95%CI) = 1.230 (1.057, 1.431), *p* = 0.008] and T2DM [OR (95%CI) = 2.106 (1.625, 2.729), *p* < 0.001]. In non-T2DM population, HbA1c was negatively correlated with ASM_BMI_ [β (95%CI) = −0.009 (−0.013, −0.005), *p* < 0.001]. The correlations only persisted in males. Furthermore, in male non-T2DM population, the association of HbA1c and ASM_BMI_ presents an inverted U-shape curve with an inflection point of HbA1c 5.2%.

**Conclusion:**

Pre-diabetes is associated with increased risk of sarcopenia. HbA1c is an independent risk factor for loss of appendicular skeletal muscle mass and sarcopenia when HbA1c greater than 5.2% in the male non-T2DM population.

## Introduction

Sarcopenia, defined as a syndrome caused by the continuous loss of skeletal muscle mass, strength, and function, has become a serious concern, especially in the elderly (1). Aging, reduction of activity, neuromuscular dysfunction, and changes in aging-related hormones (insulin, growth hormone, sex hormone, glucocorticoid) are all involved in the development of sarcopenia (2). Although sarcopenia is considered an inevitable manifestation of aging, the severity is variable depending on various factors affecting muscle metabolism (3). Lack of exercise or a sedentary lifestyle was the foremost risk factor for sarcopenia. Muscle fiber and strength decline are more obvious in individuals who lack of exercise than who are physically active (4). Age-related decreases in hormone concentrations, such as growth hormone, thyroid hormone, testosterone, and insulin-like growth factor, make for loss of muscle mass and strength (3). The motor nerve cells also decrease with age which is responsible for sending signals from the brain to the muscles to initiate movement (3, 5). Sarcopenia negatively affects the quality of life for the elderly, since it causes motor dysfunction, higher risks of falls and fractures, and disability to independent life (6). It is important to identify and prevent sarcopenia in susceptible populations.

Epidemiological studies have found that T2DM is an independent risk factor for sarcopenia. A meta-analysis revealed that the risk of sarcopenia in diabetes individuals was 109% higher than in patients without diabetes (7). Insulin resistance (IR) in skeletal muscle is reported to mediate the link between sarcopenia and T2DM (8). pre-diabetes is characterized by impaired fasting glucose or impaired glucose tolerance (9). There are few studies to clarify the association between pre-diabetes and sarcopenia. Therefore, the purpose of this study is to explore (1) whether pre-diabetes is an independent risk factor for sarcopenia and (2) the relationship between HbA1c, serum glucose, and muscle mass.

## Materials and methods

### Data sources

The NHANES (National Health and Nutrition Examination Survey) is a population-based cross-sectional survey designed to collect information on the health and nutrition status of adults and children in the United States. Most of the data in NHANES is freely accessible to researchers worldwide. This investigation pooled data from 1999 to 2006 and 2011 to 2018.

### Study population

**FIGURE 1:**
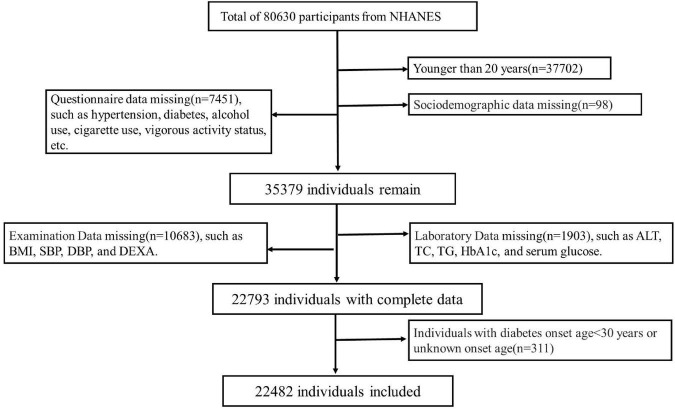
Flow chart of participants included in the study.

A total of 80,630 individuals were enrolled in NHANES from 1999 to 2006 and 2011 to 2018. Participants (*n* = 37,702) with age <20 years were excluded. Other participants were excluded due to lack of sociodemographic status (*n* = 98), questionnaire (*n* = 7451), physical examination (10,683) and laboratory data (*n* = 1,903). Individuals with diabetes onset age <30 years (*n* = 311) was also excluded to minimize the confounding from type 1 diabetes mellitus. Finally, a total of 22,482 subjects were included for data analysis. See details in Figure 1. The survey protocol was approved by the Institutional Review Board of the Centers for Disease Control and Prevention (CDC). Written consent informs were obtained from all participants.

### Study variables

#### Outcomes

The primary outcomes were sarcopenia and appendicular skeletal muscle mass (ASM) adjusted by body mass index (BMI) (ASM_BMI_). ASM was calculated by the sum of muscle mass in arms and legs measured by dual-energy X-ray absorptiometry (DXA) of the whole body by well-trained technicians. Sarcopenia was defined as ASM_BMI_ <0.512 for females and <0.789 for males. The cut-off values were based on the Foundation for the National Institutes of Health (FNIH) sarcopenia project criteria (10). Participants with weigh over 450 pounds or height over 192.5 cm were not measured by DXA.

#### Exposure

Pre-diabetes, diabetes status, HbA1c, and serum glucose were Exposures. T2DM was defined as a self-report of diabetes (ever told by a doctor that had diabetes) or HbA1c = 6.5% or serum glucose = 7.0mmol/L. Pre-diabetes was defined as HbA1c [5.7, 6.5)%, or serum glucose [5.6, 7.0) mmol/L in the non-DM population. Others were defined as having normal glucose (11). Serum glucose and HbA1c were obtained for the laboratory part in the NHANES.

#### Covariates

Covariates include five parts, which are sociodemographic variables, questionnaire data, examination data, dietary data, and laboratory data. Sociodemographic variables include age, gender, race, and education level. Hypertension (HTN), diabetes, alcohol use, cigarette use, vigorous activity status, and medicine use to lower blood glucose and cholesterol were included in the questionnaire part. Total energy intake was obtained from dietary data (24 h dietary recall interviews). Laboratory data-related variables include total cholesterol (TC), triglyceride (TG), alanine aminotransferase (ALT), serum uric acid (SUA), and Serum creatinine (SCr). Examination data-related variables include body mass index, blood pressure [systolic blood pressure (SBP) and diastolic pressure (DBP)], and total fat percentage (TFP) which was assessed by DXA of the whole body. HTN comes from three aspects: self-reported to be HTN or SBP = 140 mmHg or DBP = 90 mmHg. All data can be found in the NHANES project.^1^

### Statistical analyses

All analysis was performed by the R project^2^ and Empower Stats.^3^ NHANES sample weights were used as recommended by the National Center for Health Statistics (NCHS). *p*-Values < 0.05 were considered to be statistical significance. The baseline characteristics were shown as survey-weighted mean (95% CI) for continuous variables and survey-weighted percentage (95% CI) for categorical variables, respectively. The difference of ASM/BMI and prevalence of sarcopenia in different age groups with different diabetes status was calculated by analysis of variance. The association between diabetes status, HbA1c, or serum glucose and sarcopenia, or ASM_BMI_ were evaluated by generalized linear models to estimate theirs βs and their 95% CIs. The models were adjusted by the covariates such as age, race, gender, education level, BMI, HTN, anti-glycemic medicine and cholesterol, TG, TC, ALT, SUA, alcohol and cigarette use, TFP, and energy intake. Smooth fitting curve conducted by the generalized additive model was used to explore the non-linear relationship between HbA1c and ASM_BMI_ in non-T2DM male population, and the covariates above except for gender were also adjusted.

## Results

**FIGURE 2:**
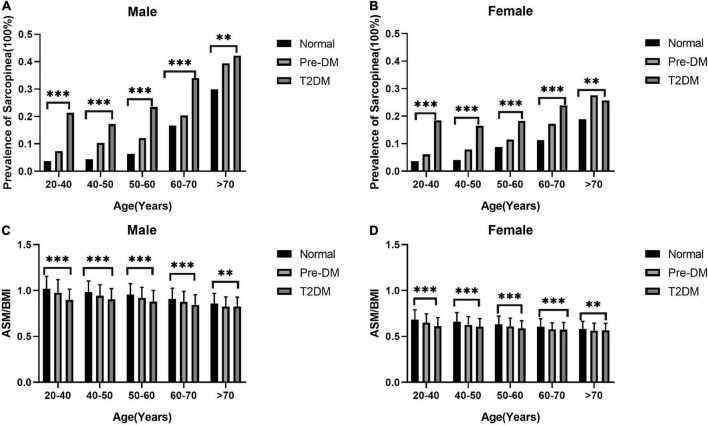
The prevalence of sarcopenia increases with age and ASM/BMI decreases. The columns in panels **(A,B)** represent the prevalence of sarcopenia in different groups of diabetes status. The columns in panels **(C,D)** represent the mean and standard deviation of ASM/BMI in different groups of diabetes status. The difference of ASM/BMI and prevalence of sarcopenia in different age groups with different diabetes status was calculated by analysis of variance. ^***^p < 0.001 and ^**^p < 0.01.

The weighted characteristics of the population included in the study was shown in Table 1. The prevalence of sarcopenia was 11.67% (2,623/22,482), however, the weighted percentage prevalence was 8.15 (7.61,8.71)% in the whole population. The prevalence of sarcopenia in normal, pre-DM, and T2DM groups were 5.41 (4.91, 5.95)%, 11.80 (10.65, 13.05)%, and 22.37 (20.26, 24.63)%, respectively (*p* < 0.001). The differences still existed in both sexes. See details in Table 2. ASM_BMI_ in normal, pre-DM, and T2DM groups were 0.82 (0.82,0.83), 0.79 (0.78,0.80), and 0.74 (0.73,0.75) (*p* < 0.001). The differences also existed in both sexes. See details in Table 3. In different grades for ages (20–40, 40–50, 50–60, 60–70, >70 years), the prevalence of sarcopenia in the pre-DM and T2DM groups was higher than in the normal group in both sexes. In different grades for ages (20-40, 40-50, 50-60,60-70, > 70 years), ASM_BMI_ in pre-DM and T2DM groups was lower than T2DM group in both sexes. See details in Figure 2.

**TABLE 1:** Baseline weighted characteristics of the population included in the study.

	Total (*n* = 22482)	Non-sarcopenia (*n* = 19859)	Sarcopenia (*n* = 2623)	*P*-value
Age (years)	42.92 (42.54,43.30)	42.08 (41.69,42.47)	52.37 (51.54,53.19)	<0.001
Age > 60 years (%)	10.89 (10.17,11.67)	9.21 (8.51,9.96)	29.84 (27.60,32.19)	<0.001
Gender (%) (male)	50.40 (49.70,51.11)	50.15 (49.35,50.94)	53.31 (50.84,55.77)	0.025
Race (%)				<0.001
Mexican American	8.33 (7.21,9.61)	7.42 (6.43,8.54)	18.56 (15.74,21.75)	
Other Hispanic	6.00 (4.91,7.30)	5.63 (4.58,6.91)	10.10 (8.09,12.54)	
Non-Hispanic White	69.25 (66.92,71.48)	69.91 (67.64,72.10)	61.74 (57.75,65.57)	
Non-Hispanic Black	10.11 (8.96,11.40)	10.78 (9.55,12.15)	2.60 (2.06,3.28)	<0.001
Other race-including multi-racial	6.32 (5.71,6.97)	6.25 (5.65,6.91)	7.01 (5.72,8.55)	<0.001
Education level = less than high school (%)	15.54 (14.53,16.60)	14.41 (13.43,15.45)	28.26 (25.92,30.73)	<0.001
BMI (kg/m^2^)	28.35 (28.18,28.51)	27.90 (27.73,28.06)	33.43 (33.06,33.81)	<0.001
HTN (%)	31.23 (30.18,32.31)	29.41 (28.38,30.46)	51.80 (48.59,54.99)	<0.001
Diabetes status				<0.001
Normal	70.52 (69.59,71.44)	72.62 (71.65,73.57)	46.81 (44.02,49.63)	
Pre-diabetes	21.39 (20.63,22.17)	20.54 (19.74,21.36)	30.97 (28.62,33.43)	
T2DM	8.09 (7.65,8.56)	6.84 (6.43,7.27)	22.21 (19.96,24.65)	
DM duration (years)	5.07 (4.74,5.40)	4.95 (4.63,5.28)	5.48 (4.73,6.23)	0.175
HbA1c (%)	5.45 (5.43,5.47)	5.41 (5.39,5.43)	5.86 (5.80,5.92)	<0.001
Serum glucose (mmol/L)	5.28 (5.25,5.31)	5.22 (5.20,5.25)	5.93 (5.82,6.05)	<0.001
Taking insulin or pills for lower glucose (%)	3.97 (3.66,4.31)	3.37 (3.07,3.71)	10.73 (9.18,12.51)	<0.001
Taking medicine for lower cholesterol (%)	9.63 (9.08,10.21)	8.74 (8.18,9.34)	19.69 (17.65,21.91)	<0.001
TC (mmol/L)	5.10 (5.08,5.12)	5.09 (5.06,5.11)	5.26 (5.20,5.32)	<0.001
TG (mmol/L)	1.66 (1.63,1.69)	1.63 (1.60,1.66)	1.99 (1.90,2.07)	<0.001
ALT (IU/L)	26.21 (25.83,26.58)	26.04 (25.64,26.43)	28.13 (27.08,29.17)	<0.001
SUA (μmol/L)	319.05 (317.55,320.54)	317.13 (315.59,318.67)	340.62 (336.22,345.03)	<0.001
SCr (μmol/L)	76.41 (75.96,76.86)	76.58 (76.14,77.01)	74.56 (73.03,76.09)	0.009
Alcohol use (%)				<0.001
Never	10.63 (9.43,11.97)	10.10 (8.85,11.51)	16.64 (14.99,18.43)	
Detoxify	14.04 (13.09,15.05)	13.16 (12.20,14.19)	23.97 (21.94,26.13)	
Current use	75.33 (73.48,77.09)	76.74 (74.82,78.55)	59.39 (56.96,61.77)	
Cigarette use (%)				<0.001
Never	53.44 (52.20,54.67)	53.48 (52.18,54.77)	52.99 (50.28,55.69)	
Detoxify	22.83 (21.97,23.72)	22.30 (21.38,23.24)	28.86 (26.57,31.25)	
Current use	23.73 (22.70,24.80)	24.23 (23.13,25.35)	18.15 (16.19,20.30)	
Vigorous activity (%)	32.32 (31.17,33.49)	33.47 (32.26,34.71)	19.29 (16.91,21.92)	<0.001
Energy (kcal)	2260.89 (2243.04,2278.74)	2289.15 (2269.22,2309.07)	1942.25 (1901.78,1982.73)	<0.001
ASM_BMI_	0.81 (0.81,0.81)	0.83 (0.82,0.83)	0.62 (0.61,0.62)	<0.001
Sarcopenia (%)	8.15 (7.61,8.71)	0	100.00	<0.001
TFP (%)	33.43 (33.23,33.62)	32.81 (32.61,33.01)	40.38 (40.04,40.73)	<0.001

For continuous variables: survey-weighted mean (95% CI), *p*-value was by survey-weighted linear regression. For categorical variables: survey-weighted percentage (95% CI), *p*-value was by survey-weighted Chi-square test. *P* indicated the difference between sarcopenia and non-sarcopenia groups. BMI, body mass index; HTN, hypertension; T2DM, type 2 diabetes mellitus; HbA1c, glycohemoglobin; TC, total cholesterol; TG, triglyceride; ALT, alanine aminotransferase; SUA, serum uric acid; SCr, serum creatinine; ASM, appendicular skeletal muscle mass. ASM_BMI_ = ASM/BMI. TFP = total fat percentage.

**TABLE 2:** The prevalence of sarcopenia in different diabetes status populations included in the study.

	Normal	Pre-DM	T2DM	*P*-value
Sarcopenia (%)	5.41 (4.91,5.95)	11.80 (10.65,13.05)	22.37 (20.26,24.63)	<0.001
**Subgroup analysis**				
Male				<0.001
Sarcopenia (%)	5.36 (4.77,6.02)	12.30 (10.83,13.94)	24.72 (21.61,28.11)	
Female				<0.001
Sarcopenia (%)	5.45 (4.78,6.22)	11.20 (9.77,12.80)	19.70 (16.90,22.83)	

For categorical variables: survey-weighted percentage (95% CI), *p*-value was by survey-weighted Chi-square test.

**TABLE 3:** The ASM_BMI_ in different diabetes status populations included in the study.

	Normal	Pre-DM	T2DM	*P*-value
ASM_BMI_	0.82 (0.82,0.83)	0.79 (0.78,0.80)	0.74 (0.73,0.75)	<0.001
**Subgroup analysis**				
**Male**				
ASM_BMI_	0.99 (0.99,0.10)	0.94 (0.93,0.94)	0.88 (0.87,0.89)	<0.001
**Female**				
ASM_BMI_	0.66 (0.66,0.67)	0.62 (0.61,0.62)	0.59 (0.58,0.60)	<0.001

For continuous variables: survey-weighted mean (95% CI), *p*-value was by survey-weighted linear regression.

### The association between sarcopenia, ASM_BMI_, and diabetes status

After fully adjusting for multiple covariates, the pre-diabetes [OR (95%CI) = 1.230 (1.057, 1.431), *p* = 0.008] and T2DM [OR (95%CI) = 2.106 (1.625, 2.729), *p* < 0.001] are independent risk factor for sarcopenia in the whole population. In both sexes, pre-diabetes and T2DM are risk factors for sarcopenia. Similarly, both pre-diabetes and T2DM were associated with lower ASM_BMI_ than the normal group [β (95%CI) = −0.007 (−0.010, −0.004), *p* < 0.001 for pre-diabetes, and β (95%CI) = −0.021 (−0.027, −0.016), *p* < 0.001for TDM]. In males, pre-diabetes and T2DM were associated with lower ASM_BMI_. In females, T2DM, not pre-diabetes, was associated with lower ASM_BMI_. See details in Table 4.

**TABLE 4:** Association between ASM_BMI_, sarcopenia, and diabetes status.

Exposure		OR (95%CI) *p*-value	β (95%CI) *p*-value
		**Sarcopenia as outcome**	**ASM_BMI_ as outcome**
	**Normal**	**Reference**	**Reference**
Total	Pre-diabetes	1.230 (1.057, 1.431)0.008	−0.007 (−0.010, −0.004) < 0.001
	T2DM	2.106 (1.625, 2.729) < 0.001	−0.021 (−0.027, −0.016) < 0.001
Male	Pre-diabetes	1.215 (1.014,1.455)0.038	−0.009 (−0.014, −0.005) < 0.001
	T2DM	2.268 (1.546,3.327) < 0.001	−0.028 (−0.038, −0.019) < 0.001
Female	Pre-diabetes	1.253 (1.013,1.551)0.040	−0.003 (−0.007,0.001)0.117
	T2DM	1.950 (1.309,2.904)0.001	−0.008 (−0.015, −0.001)0.021

Adjusted for age, gender, race, BMI, HTN, alcohol use, cigarette use, vigorous activity status, education level, TC, TG, SUA, SCr, ALT, TBP, insulin or pills use to lower glucose, taking medicine to lower cholesterol, and total energy intake. Gender was not adjusted for in the subgroup analysis. T2DM, type 2 diabetes mellitus. ASM_BMI_ = ASM/BMI.

### Association between HbA1, serum glucose and ASM_BMI_

After fully adjusting for multiple covariates, ASM_BMI_ was negatively correlated with HbA1c [β (95%CI) = −0.004 (−0.006, −0.002), *p* < 0.001] in the whole population. The correlation existed only in males [β (95%CI) = −0.007 (−0.009, −0.004), *p* < 0.001]. After fully adjusting for multiple covariates, ASM_BMI_ was negatively correlated with HbA1c in the non-T2DM population. After stratified by gender, the correlation only existed in males. After fully adjusting for multiple covariates, serum glucose was negatively correlated with ASM_BMI_ [β (95%CI) = −0.001 (−0.002, −0.001), *p* = 0.003]. The correlation only existed in males. The correlation disappeared in T2DM and non-DM. see details in Table 5.

**TABLE 5:** Association of HbA1c, and serum glucose with ASM_BMI_.

Exposure	β (95%CI) *p*-value		
	**Total**	**T2DM**	**Non-DM**
**HbA1c (%)**			
Total	−0.004 (−0.006, −0.002) < 0.001	0.000 (−0.002,0.002)0.950	−0.009 (−0.013, −0.005) < 0.001
Male	−0.007 (−0.009, −0.004) < 0.001	0.000 (−0.003,0.004)0.872	−0.015 (−0.021, −0.009) < 0.001
Female	0.000 (−0.002,0.002)0.985	0.000 (−0.002,0.003)0.796	−0.001 (−0.006, 0.004)0.717
**Serum glucose (mmol/L)**			
Total	−0.001 (−0.002, −0.001)0.003	−0.000 (−0.001,0.001)0.513	−0.001 (−0.003,0.002)0.683
Male	−0.002 (−0.003, −0.000)0.010	−0.000 (−0.002,0.002)0.828	0.000 (−0.003,0.004)0.862
Female	−0.001 (−0.002,0.000)0.219	−0.001 (−0.002,0.001)0.286	−0.001 (−0.004,0.003)0.670

Adjusted for age, gender, race, BMI, HTN, alcohol use, cigarette use, vigorous activity status, education level, TC, TG, SUA, SCr, ALT, TBP, taking insulin or pills for lower glucose, taking medicine to lower cholesterol, and total energy intake. DM duration was also adjusted for in the T2DM group, and taking insulin or pills for lower glucose was not adjusted in non-DM group. Gender was not adjusted for in the subgroup analysis depending on the sexes. T2DM, type 2 diabetes mellitus; HbA1c, glycohemoglobin; ASM, appendicular skeletal muscle mass. ASM_BMI_ = ASM/BMI.

**FIGURE 3:**
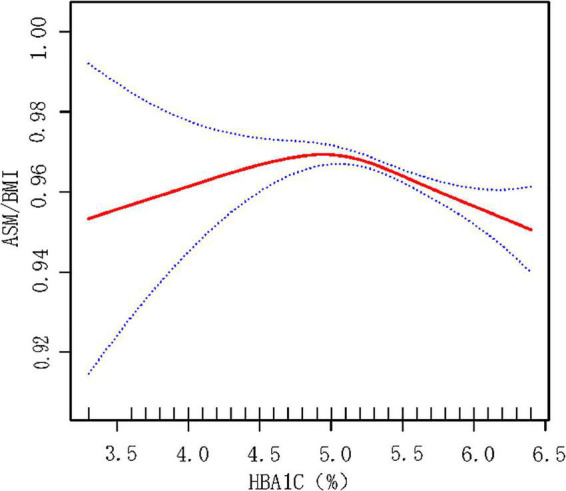
Smooth fitting curve reflecting the relationship between HbA1c and ASM_BMI_ in male non-T2DM population. Adjusted for age, race, BMI, HTN, alcohol use, cigarette use, vigorous activity status, education level, TC, TG, SUA, SCr, ALT, TBP, taking medicine for lowing cholesterol, and total energy intake. HbA1c below 5.2% may indicate malnutrition, which is associated with less muscular mass. HbA1c over 5.2% may indicate over energy storage and abnormal glycemia metabolism. ASM, appendicular skeletal muscle mass. ASM_BMI_ = ASM/BMI.

Smooth fitting curve conducted by the generalized additive model was used to explore the non-linear relationship between HbA1c and ASM_BMI_ in non-T2DM male population. There is an inversely U-shaped relationship between HbA1c and ASM_BMI_ (Figure 3). Two piecewise models found an inflection point at 5.2% in HbA1c. Before the inflection point, the HbA1c seems positively correlated with ASM_BMI_ but without statistical significance (*p* = 0.590). After the inflection point, HbA1c was negatively correlated with ASM_BMI_ (*p* < 0.001). The log-likelihood ratio between a standard model and two piecewise models was of statistical significance (*p*-value < 0.001). See details in Table 6.

**TABLE 6:** Two piecewise model for relation between hemoglobin and ASM_BMI_ in non-T2DM male population.

Outcome	β (95%CI) *p*-value
Fitting by a standard linear model	−0.015 (−0.021, −0.009) < 0.001
Fitting by two-piecewise linear models	
Inflection point (%)	5.2
HbA1c < inflection point	0.003 (−0.008,0.014)0.590
HbA1c > inflection point	−0.028 (−0.036, −0.019) < 0.001
Log-likelihood ratio	<0.001

Adjusted for age, race, BMI, HTN, alcohol use, cigarette use, vigorous activity status, education level, TC, TG, SUA, SCr, ALT, TBP, taking medicine for lowing cholesterol, and total energy intake. T2DM, type 2 diabetes mellitus; HbA1c, glycohemoglobin; ASM, appendicular skeletal muscle mass. ASM_BMI_ = ASM/BMI.

## Discussion

This study firstly revealed that pre-diabetes was associated with loss of appendicular skeletal muscle (ASM) mass, and it increases risk of sarcopenia in US adults. It suggests that decreasing ASM occurs in the early stage of abnormal glucose metabolism and more attention should be paid to identify and prevent sarcopenia. HbA1c was the independent risk factor for sarcopenia in the male non-DM population and ASM tended to decrease when the HbA1c exceed 5.2%.

The prevalence of sarcopenia in T2DM can be as high as 30% (12). The strong relationship between sarcopenia and glycometabolism can be explained by several mechanisms. First, insulin plays an anabolic role in skeletal muscle. The impairment in insulin action may intervene protein synthesis and decrease muscle mass and strength (13). Second, chronic hyperglycemia promotes the accumulation of advanced glycosylation end products (AGEs) in skeletal muscle which is related to the reduction of grip strength, leg extension strength, and walking speed (14). Third, increasing inflammatory cytokines may contribute to the reduction of muscle mass and strength (15). Forth, complications of T2DM are also related to sarcopenia. Chronic renal insufficiency is closely related to sarcopenia. Diabetic retinopathy may lead to decreased exercise, and then muscle mass decline. Diabetic peripheral neuropathy also can impair muscle strength (12). Effects of pre-diabetes on muscles may share the same mechanisms with diabetes such as insulin resistance, the increase of inflammatory cytokines, and accumulation of AGEs (16, 17).

Some studies have explored the relationship between pre-diabetes and grip strength or muscle mass. A study conducted by Shan Hu etc., found greater grip strength was associated with lower incidence of pre-diabetes in Chinese adults (18). A prospective cohort study showed that a higher level of handgrip strength promise a lower risk of pre-diabetes [HR 95% CI = 0.38 (0.21–0.71)] among adults in 2 years follow-up (19). Besides, Srikanthan found that every 10% increase in skeletal muscle index (SMI) was associated with 12% reduction in pre-or overt diabetes (20). Few studies had been conducted to investigate the correlation between pre-diabetes and sarcopenia. A recent study conducted in Japan revealed increasing sarcopenia rate in pre-diabetic older men, but not women (21). Focusing on the whole US population, our study found that pre-diabetes was an independent risk factor for sarcopenia.

The results of association between HbA1c and loss of ASM or sarcopenia was not consistent. It was reported that a higher HbA1c level (> 8%) was associated with lower skeletal muscular index (SMI) (OR = 5.35, *p* < 0.001) compared with lower HbA1c (<6%) (22). However, another study found that male patients with sarcopenia had lower HbA1c than those without sarcopenia (6.5 vs. 7.1%) (23). Our investigation focused on the glucose metabolism and ASM in the non-T2DM male population. The serum glucose showed statistical significance with loss of ASM only in the whole population [β (95%CI) = −0.001 (−0.002, −0.001), *p* = 0.003]. However, the association did not exist in both sexes and T2DM or not. HbA1c was negatively correlated with ASM_BMI_ [β (95%CI) = −0.015 (−0.021, −0.009), *p* < 0.001] in males. Furthermore, a non-linear relationship between HbA1c and ASM_BMI_ was found that when HbA1c exceeds 5.2%, the skeletal muscle mass tends to decline. It is presumed that HbA1c below 5.2% may indicate a state of malnutrition, which may cause loss of skeletal muscles. It was reported that HbA1c levels were significantly decreased in young adult malnourished patients without disease (24). Low concentrations of HbA1c was a significant predictors of poor nutritional status (defined by ≥1 micronutrient deficiency in blood of vitamins B6, B12, and C; selenium; or zinc) (25). Although previous studies have shown that people with lower HbA1c levels have higher prevalence of malnutrition, weight loss and other complications (26), our research couldn’t reach a definite conclusion since there is no statistically significant difference for the relationship between ASM/BMI and HbA1c when HbA1c <5.2%. On the other hand, HbA1c over 5.2% indicates a state of insulin resistance and poorly controlled diabetes, which may cause skeletal muscle decrease by the mechanism mentioned above.

Our study found that the effects of pre-diabetes on ASM_BMI_ seemed more remarkable in males than in female [β (95%CI) = −0.009 (−0.014, −0.005) in males vs. −0.003 (−0.007, 0.001) in females]. Similarly, the effect of HbA1c on ASM_BMI_ was detected only in males. Such a gender differences were also seen in other investigations. A cohort study, the English Longitudinal Study of Ageing, shown that diabetes was a risk factor for sarcopenia only in males, not in females [OR95%CI = 2.43 (1.50, 3.95) in males vs. 1.49 (0.83, 2.68) in females] (27). A meta-analysis shown a OR of sarcopenia in T2DM men was 1.72 (95%CI: 1.1–2.69) and was 1.46 (95%CI: 0.94–2.25) in women (28). An Asian study conducted in Japan reported the sex different for pre-diabetes to sarcopenia existed [OR95%CI: 2.081 (1.031–4.199) for men vs. 1.036 (0.611–1.757) for women]. The gender difference might be explained by (1) the muscle mass decline with age was more remarkable in males than in females (29) and (2) ASM mass is greatly influenced by other factors such as testosterone level, which decreased in men with T2DM and obesity.

Our research has some limitations. First, a cross-sectional designed study cannot identify the causal relationship between sarcopenia and diabetes status. Less active life style and decreasing ASM may lead to decreasing insulin sensitivity and diabetes, which could also explain our results. Second, sarcopenia was defined from ASM_BMI_ in this study. Grip strength and walking speed, reflecting the quality of ASM, were not taken into consideration due to data limitation.

## Conclusion

Both pre-diabetes and T2DM are associated with higher risk of loss of ASM and sarcopenia. In non-T2DM male population, increasing HbA1c is associated with the decrease ASM_BMI_ and increase risk of sarcopenia. The inverted U-shaped relationship between HbA1c and ASM_BMI_ reminds us malnutrition or too much energy store may intend to loss of appendicular skeletal muscles.

## Data availability statement

The raw data supporting the conclusions of this article will be made available by the authors, without undue reservation.

## Ethics statement

The studies involving human participants were reviewed and approved by the Ethics Review Board of the National Center for Health Statistics approved all NHANES protocols. The patients/participants provided their written informed consent to participate in this study.

## Author contributions

SL contributed to the data collection, statistical analysis, and writing and revising of the manuscript. JM and WZ supervised the study and contributed to the polishing and reviewing of the manuscript. All authors contributed to the article and approved the submitted version.

## Footnotes

1
http://www.cdc.gov/nchs/nhanes/

2
http://www.Rproject.org

3
http://www.empowerstats.com
